# COVID-19 and PIMS—Two Different Entities, but the Same Trigger

**DOI:** 10.3390/children9091348

**Published:** 2022-09-04

**Authors:** Lorena Elena Meliț, Cristina Oana Mărginean, Tudor Fleșeriu, Anca Meda Văsieșiu, Dana Valentina Ghiga, Ana-Maria Roxana Koller

**Affiliations:** 1 Department of Pediatrics I, “George Emil Palade” University of Medicine, Pharmacy, Science, and Technology of Târgu Mureș, Gheorghe Marinescu Street No. 38, 540136 Târgu Mureș, Romania; 2 Department of Infectious Disease, County Clinical Hospital Târgu Mureș, Gheorghe Doja Street No. 89, 540394 Târgu Mureș, Romania; 3 Department of Infectious Disease, “George Emil Palade” University of Medicine, Pharmacy, Science, and Technology of Târgu Mureș, Gheorghe Marinescu Street No. 38, 540136 Târgu Mureș, Romania; 4 Department of Scientific Medical Research Methodology, “George Emil Palade” University of Medicine, Pharmacy, Science and Technology from Târgu Mureș, Gheorghe Marinescu Street No. 38, 540136 Târgu Mureș, Romania; 5 Department of Pediatrics I, Emergency County Clinical Hospital Târgu Mureș, Gheorghe Marinescu Street No. 50, 540136 Târgu Mureș, Romania

**Keywords:** COVID-19, PIMS, children

## Abstract

COVID-19 and PIMS represent two novel pathologies that have challenged the medical world during the last two years on account of their being very similar, but yet very different. *Our aim* was to comparatively assess children with SARS-CoV-2 infection and PIMS in terms of symptoms, clinical findings, laboratory parameters, echocardiography, and evolution. Our retrospective study included 46 children with COVID-19 (group 1), and 20 children with confirmed PIMS (group 2). We found no significant differences in terms of age, gender, and originating area between the two groups. We noticed that fever was significantly more common in the PIMS group as compared to COVID-19 group (*p* = 0.0217). In terms of laboratory parameters, increased bilirubin and creatinine were significantly more frequent in children with COVID-19 (*p* = 0.0064/*p* = 0.0064), while hypoalbuminemia and elevated ESR were significantly more common in those with PIMS (*p* < 0.0001/*p* = 0.0127). Moreover, prognosis parameters such as D-dimers, NT-proBNP, and CK-MB were also found to be significantly higher in the PIMS group as compared to COVID-19 group (*p* = 0.0003/*p* = 0.0182/*p* = 0.0007). In terms of complications, most were identified in PIMS group, among which cardiac and liver impairment along with dehydration were significantly more common in children diagnosed with PIMS as compared to those detected with COVID-19. Similarly, children with PIMS had a significantly higher chance to have pathological echocardiography changes. Although difficult, the distinction between COVID-19 and PIMS is crucial for the patient’s long-term outcome.

## 1. Introduction

It has been more than two years since the beginning of the COVID-19 pandemic and much has been learnt about this pathology, but there still remain several gaps in knowledge in this field. Although initially children were considered to be spared from developing this condition or to remain healthy carriers, they were later proven to manifest even severe life-threatening forms of this disease [1]. As of March 2022, in Romania, pediatric cases confirmed with COVID-19 accounted for 12% of all cases [2]. Indeed, children are less susceptible than adults in terms of COVID-19, most likely due to a combination of factors, including the immaturity of the immune system in this age group [3]; viral co-infection, frequent in children, due to competition and interaction between viruses [4]; improper maturation, distribution, and functioning of the angiotensin-converting enzyme receptor, essential for the adherence of SARS-CoV-2 virus [5]; or the phenomenon of ‘trained immunity’ as a result of Bacillus Calmette–Guérin vaccine which triggers heterologous immunity to other pathogens involving cells of the innate immune system like monocytes, macrophages, and epithelia [6].

The diagnosis of SARS-CoV-2 infection in children is frequently burdened by the absence of symptoms or the presence of non-specific symptoms in pediatric patients, which commonly overlap with those of influenza including fever, cough, sneezing, a sore throat, myalgia, and fatigue [7]. Moreover, based on the findings of a large study which included 2135 children, up to 13% of those who were confirmed with this infection presented no symptoms [7]. A similar incidence of asymptomatic carriers of SARS-CoV-2 was indicated by a study from China which underlined that 15.8% of the 171 children detected with this infection manifested no symptoms [8]. Severe forms of COVID-19 seem to be less common in children as compared to adults or other previously reported severe acute respiratory syndromes. Thus, a study which compared these two types of SARS pointed out severe discrepancies in terms of symptoms and evolution between children with SARS 2003 and the current SARS-CoV-2 infection [9]. The authors found no asymptomatic cases during the infection from 2003 when compared to 29.1% of the patients diagnosed with SARS-CoV-2 infection who presented no symptoms at admission. Moreover, fever was commonly observed in patients with SARS 2003, while only a half (51.3%) of those detected with SARS-CoV-2 presented fever. Similarly, only 4.7% of COVID-19 children required oxygen supplementation during admission, as compared to 18.6% of those with SARS 2003 [9]. Gastrointestinal symptoms such as loss of appetite, nausea, vomiting, diarrhea, abdominal pain, or gastrointestinal bleeding are also frequently reported in COVID-19, with children being reported in almost 80% of the cases [10]. Although laboratory parameters are not specific in these patients, a review based on 66 children diagnosed with this condition revealed normal leucocyte count in 69.2% of the patients, 6% being found with neutropenia, 4.6% with neutrophilia in 4.6%, and 3% with lymphopenia [11]. The same authors indicated that the level of C-reactive protein was above the normal limit in 13.6% of these cases.

Pediatric inflammatory multisystem syndrome (PIMS) is a severe, life-threatening condition which occurs in children recently diagnosed with SARS-CoV-2 infection independently of the form of infection [12,13,14,15]. Thus, this syndrome might also occur in cases with mild forms of COVID-19 and even in those with no symptoms during the infection. Complex combined diagnostic criteria were defined by several experts of the World Health Organization, The Royal College of Pediatrics and Child Health, as well as of Centers for Disease Control and Prevention involving evidence of COVID-19 (antigen test, real-time polymerase chain reaction or serology positive), no other proof of infection, fever, conjunctivitis, hypotension or shock, cardiac or other organ involvement (respiratory, dermatological, renal, neurological), acute abdominal pain or other acute gastrointestinal impairment, and elevated inflammatory markers [16,17,18]. In fact, this condition is rather similar to toxic shock syndrome or Kawasaki disease [16,19]. In terms of laboratory parameters, children with PIMS were commonly found with increased inflammatory markers, consisting of high C-reactive protein (CRP) in 94% of the cases, but also neutrophilia in 83% or lymphopenia in 50% of the patients [20]. Moreover, cardiac involvement was indicated based on increased troponin in 68% of the cases, as well as brain natriuretic peptide (proBNP) in 77% of them [20].

Based on the similarities such as fever, gastrointestinal symptoms, neutrophilia, lymphopenia, or elevated C-reactive protein, but also the differences between these two clinical entities, such as more severe clinical forms of PIMS or the increased likelihood of elevated C-reactive protein and cardiac involvement in children with PIMS, it would be extremely useful to compare the presenting concerns, the clinical findings, and laboratory parameters, as well as the clinical course between children with SARS-CoV-2 infection and patients with PIMS in order to delineate the boundaries between these two conditions and to identify the cases that carry a high risk for severe complications as soon as possible.

*Our aim* was to assess by comparison children with SARS-CoV-2 infection and PIMS in terms of symptoms, clinical findings, laboratory parameters, echocardiography, and evolution.

## 2. Materials and Methods

### 2.1. Study Design

We performed a correlational cross-sectional retrospective study on children admitted to the Pediatrics Clinic 1 Târgu Mureş, Romania, between May 2020 and April 2022. The children were divided into two groups: group 1—children diagnosed with COVID-19 and group 2—children diagnosed with PIMS. The inclusion criteria for group 1 consisted of a confirmed SARS-CoV-2 infection based on a positive RT-PCR test, while for group 2 patients who fulfilled the criteria of PIMS and have a documented SARS-CoV-2 infection based on a positive antigen test, RT-PCR, or serology (positive IgM and/or IgG anti-SARS-CoV-2). Patients with incomplete clinical and paraclinical data were excluded from the study. All the data were collected from the clinical charts of the patients.

The children included in this study benefited from a thorough anamnesis according to the statements of their parents/caregivers and clinical exam. We analyzed all the presenting clinical symptoms the patients experienced in both groups. Several laboratory parameters were assessed at the time of admission in both groups, i.e., complete blood count, inflammatory biomarkers (erythrocyte sedimentation rate—ESR, CRP, interleukin—IL-6, ferritin), liver function parameters (aspartate aminotransferase—AST, alanine aminotransferase—ALT, total bilirubin), renal function parameters (urea, creatinine), electrolytes (sodium—Na, potassium—K), total proteins, albumin, lactate dehydrogenase (LDH), D-dimers, creatine kinase (CK), and cardiological parameters (NT-proBNP, high sensitive troponin, CK-MB). Moreover, we assessed the complications and the treatment in both groups.

### 2.2. Ethical Statements

The research was approved by the Ethics Committee of the University of Medicine, Pharmacy, Sciences and Technology in Târgu Mureș (No 1738/12 May 2022). The study was performed according to the principles of the Helsinki Declaration.

### 2.3. Statistical Analysis

The statistical analysis included both descriptive (frequency, percentage, mean, median, standard deviation) and elements of inferential statistics. We applied the Shapiro–Wilk test for identifying the distribution of the analyzed data series. The t-Student test was used for comparing the unpaired data, while the Mann–Whitney assessed the comparison of medians. We also applied the Chi squared test and Fisher test for determining the association of qualitative variables. To quantify the relationship between more predictor variables and the outcome variable we performed logistic regression. The significance threshold was chosen for a *p*-value of 0.05. The statistical analysis was performed with the EpiInfo program.

## 3. Results

The Demographic Analysis of the Sample

Our study included 46 children with COVID-19 (group 1), and 20 children with confirmed PIMS (group 2). We found a similar mean age between the 2 groups, 6.390 ± 5.710 years for COVID-19 group and 6.600 ± 4.710 years for PIMS group (*p* = 0.40).

We found no significant association between the originating area or gender and COVID-19 or PIMS (*p* = 0.58/*p* = 0.28) (Table 1).

The symptoms in the two group were similar, including nasal obstruction, dysphonia, cough, diarrhea, vomiting, abdominal pain, and myalgia (*p* = 0.16/*p* = 0.26/*p* = 0.99/*p* = 0.99/*p* = 0.16/*p* = 0.28/*p* = 0.16), except for fever which was significantly more frequent in patients with PIMS (*p* < 0.05) (Table 2).

Regarding the hematological and biochemical parameters of the two study groups, we obtained significant higher value for total bilirubin in the COVID-19 group (0.6848 ± 0.6556 mg/dl) versus PIMS group (0.3115 ± 0.1514 mg/dL) (*p* < 0.05), as well as for creatinine (0.5848 ± 0.6345 mg/dL versus 0.4030 ± 0.1589 mg/dL, *p* < 0.05). Contrariwise, the value of ESR was significantly higher in children with PIMS (38.25 ± 35.54 mm/h) when compared to those with COVID-19 (17.39 ± 16.33 mm/h), *p* < 0.05. Similarly, hypoalbuminemia was significantly more common in the PIMS group as compared to COVID-19 (*p* < 0.05) (Table 3).

We obtained higher values for ALT, AST, urea, Na, K, total protein, LDH, leukocytes, Hgb, lymphocytes, and ferritin in the COVID-19 group than the PIMS group, but without statistical significance. Contrariwise, we obtained lower values for Htc, VEM, platelets, neutrophils, monocytes, eosinophils, and CRP in the COVID-19 group than the PIMS group, but also without statistical significance (Table 3).

Analyzing the association between inflammatory laboratory parameters and COVID-19 infection or PIMS we observed that higher values of D-dimers are more frequent associated with PIMS (OR = 8.50, 95% CI: 2.541–28.435, *p* < 0.05). Additionally, our findings revealed that patients with PIMS had higher chances to present increased levels of NT-proBNP (OR = 4.111, 95%.CI: 1.315–12.854, *p* < 0.05) and CK-MB (OR = 8.148, 95% CI: 2.381–27.879, *p* < 0.05). Instead, we found no significant differences regarding the associations between IL-6, hs-cTnI or CK and COVID-19 infection or PIMS (*p* = 0.71/*p* = 0.58/*p* = 0.74) (Table 4).

According to the logistic regression, we noticed that only D-dimers and CK-MB remained significant predictors of PIMS (*p* < 0.05) (Table 5 and Table 6).

Complications were more frequent in children with PIMS. Thus, cardiac impairment was significantly more common in the PIMS group when compared to the COVID-19 group (*p* < 0.05). Similarly, liver impairment and dehydration syndrome were also more common in children with PIMS as compared to those with COVID-19 (*p* < 0.05/*p* < 0.05). All the assessed complication were detailed in Table 7.

Regarding the echocardiography examination, we observed that patients with PIMS had higher chances of associated echocardiography changes involving pericarditis, myocarditis, left ventricular dysfunction, or coronary dilation, as compared to patients with COVID-19 infection (OR = 4.778, IC (95%): 1.016–22.459, *p* < 0.05).

## 4. Discussion

Our study underlined that among the clinical symptoms, fever seems to be the most relevant in terms of differentiating COVID-19 from PIMS, since it was significantly more common in children with PIMS. In terms of laboratory parameters, our analysis revealed that total bilirubin and creatinine levels were significantly higher in COVID-19 patients, whereas patients with PIMS were more commonly found with hypoalbuminemia and increased ESR, suggesting that the systemic inflammation is more severe in this group. The increased severity of PIMS when compared to COVID-19 was also emphasized by the higher likelihood of thromboembolic events due to the significantly elevated D-dimers, but also by the higher chance to develop cardiac impairment based on the significantly higher levels of NT-proBNP and CK-MB in the setting of PIMS. The latter was confirmed by echocardiography which revealed pathological findings to be significantly more frequent in PIMS patients.

COVID-19 is a relatively new pathology and multiple knowledge gaps related to the pathogenesis, clinical picture, and long-term complications require close monitoring and further longitudinal studies. COVID-19 and PIMS represent two clinical entities with similarities and differences, which in fact have the same trigger, SARS-CoV-2 infection. COVID-19 in children is definitely less severe than in adults, while PIMS represents a life-threatening condition regardless of the patient’s age. Therefore, the distinction between these two clinical entities might be life-saving. In terms of gender and age, PIMS usually affects children above the age of 6 years and tends to be more frequent in boys [21]. Although without statistical significance, our study also indicated the male gender to be more frequently diagnosed with PIMS, and the mean age of PIMS children was also above the age of 6 years. Unfortunately, the symptoms of both COVID-19 and PIMS frequently overlap, especially in terms of gastrointestinal symptoms and fever [22,23]. In fact, fever is a mandatory symptom according to the diagnostic criteria of PIMS, but it seems that only half of COVID-19 children develop fever [8,16,17,18]. A recent meta-analysis which reviewed 98 publications also underlined fever and gastrointestinal symptoms to be the most common symptoms in children with PIMS [24]. Similar findings were reported in the review of Patel J et al. [21]. These findings were confirmed by our study which proved that fever was significantly more common in patients with PIMS when compared to those with COVID-19.

In terms of laboratory parameters, inflammatory biomarkers such as CRP or ESR are extremely useful for assessing the severity of both COVID-19 and PIMS and to monitor their clinical course. Most of the pediatric studies pointed out that CRP levels are increased in patients with PIMS [25,26,27,28]. Nevertheless, our study failed in finding a significant association between this parameter and PIMS, suggesting that it is not a reliable indicator for distinguishing acute COVID-19 from PIMS. Contrariwise, ESR might be useful for the differential diagnosis of these two entities since we noticed that it is significantly more increased in children with PIMS when compared to those with COVID-19. It was also underlined that children with PIMS commonly associate lymphopenia, neutrophilia, anemia, as well as increased lactate dehydrogenase and ferritin [25,27,28,29]. Nevertheless, our study found no significant differences between children with PIMS and those with COVID-19 in terms of these parameters suggesting that they are not reliable for distinguishing these two clinical entities. Taking into account the great similarities between Kawasaki disease and PIMS, it is worth mentioning that studies performed on patients with Kawasaki disease proved that hypoalbuminemia might be associated with adverse coronary outcomes [30]. Thus, most of the PIMS patients have elevated levels of ESR and CRP, ferritin, D-dimers, LDH, and procalcitonin, but there may also be associated neutrophilia, thrombocytopenia and hypoalbuminemia [21]. Our study proved that patients with PIMS present a significantly higher level of albumin in comparison to COVID-19 group. Moreover, the level of creatinine was significantly lower in PIMS group when compared to COVID-19, suggesting potential renal impairment, most often of prerenal cause due to dehydration. Contrariwise, the level of bilirubin was significantly higher in the COVID-19 group in comparison to children with PIMS.

COVID-19 associated coagulopathy is a severe complication triggered by this infection, resulting in a high risk for arterial, venous, or microvascular thrombosis [31]. Thus, several thrombotic events were reported in patients with COVID-19 such as pulmonary embolism, ischemic stroke, deep venous thrombosis, myocardial infarction, and systemic arterial thrombosis [31]. Moreover, COVID-19 pneumonia non-survivors were encountered with abnormal coagulation parameters among which prolonged prothrombin time, increased D-dimers levels, increased fibrinogen degradation products and activated thromboplastin time, suggesting that the impairment of these parameters might define the poor outcome of these patients [32]. In addition, these changes in coagulation parameters were proven to persist after hospitalization in COVID-19 survivors [32], suggesting the long-term risk for thrombotic events even after the acute period of COVID-19. Thus, PIMS might be defined as the period of thrombotic events, fact that was also proven by our study since we encountered in the PIMS group one case with ischemic stroke. All these hypotheses were confirmed by autopsies performed on patients diagnosed with COVID-19 who associated increased D-dimers, ferritin, and fibrinogen levels along with a prolonged prothrombin time revealing small and firm thrombi within the lung parenchyma, as well as diffusely edematous lung parenchyma with peripheral hemorrhages [33]. In addition, it was proven that increased levels of D-dimers might be defined as a reliable predictor of thrombotic events and bleeding resulting in critical illness and even death [34]. Despite the fact that we encountered increased levels of D-dimers in both our groups, we noticed that the likelihood of this parameter to be increased was significantly higher in children with PIMS as compared to those with COVID-19 according to both univariate and logistic regression analyses.

A recent meta-analysis that compared children with PIMS with COVID-19 patients pointed out that when compared to non-severe COVID-19 patients, PIMS patients presented lower absolute lymphocyte count along with more elevated absolute neutrophil count, D-dimers, and CRP levels [35]. Contrariwise, the authors pointed out that when compared to severe forms of COVID-19, PIMS patients had lower platelets count and LDH levels, as well as higher ESR levels. Our study confirmed these findings in terms of ESR and D-dimers levels, highlighting their utility in differentiating these two pathologies.

Cardiological complications due to SARS-CoV-2 infection represent one of the life-threatening events related to this infection. Several parameters were revealed to be useful in assessing or predicting myocardial involvement during or after COVID-19, such as troponin, NT-proBNP [25,29], or CK-MB. Thus, increased troponin and NT-proBNP levels are not uncommon in the setting of cardiac involvement indicating myocardial injury. Therefore, most PIMS patients have elevated levels of ESR and CRP, ferritin, D-dimers, LDH, and procalcitonin, but they may also have associated neutrophilia, thrombocytopenia and hypoalbuminemia [21]. Similarly, our study pointed out that, in children with PIMS there is an associated and significantly higher chance of presenting increased levels of both NT-proBNP and CK-MB. Nevertheless, after applying the logistic regression, only CK-MB remained a significant predictor of PIMS. Moreover, we noticed that cardiac impairment was significantly more common in children with PIMS as compared to those with COVID-19. Still, the studies reported in the literature underlined the complete resolution of left ventricular dysfunction, commonly seen in the setting of SARS-CoV-2 infection, along with a decrease in these biomarkers after intravenous immunoglobulin administration [36]. Therefore, a French study highlighted that of all children who were detected with severe left ventricular dysfunction and a decreased ejection fraction, 71% experienced a complete resolution, emphasizing that myocardial edema is likely to be responsible for these dysfunctions and not necrosis as reported in adults [26]. Our findings support the previously mentioned statements, since cardiac impairment was significantly more common in children with PIMS that in those with COVID-19, but none of the children included in either of the two presented a fatal outcome. In addition, we noticed that, in children, PIMS had a significantly higher chance of detection with echocardiography pathological findings when compared to those with COVID-19.

Based on our findings, we might state that each patient with suspected COVID-19 or PIMS should benefit from a through anamnesis and clinical exam. Further diagnostic process should consist in the assessment of complete blood count, inflammatory biomarkers (CRP, ESR, Il-6, ferritin), as well as LDH, renal, and liver functional parameters, albuminemia, and total protein. Moreover, D-dimers are extremely important in order to predict and prevent thromboembolic event. The assessment of cardiologic parameters (troponin, CK-MB, and NT-proBNP) along with echocardiography as being of major importance, especially in the diagnostic management of PIMS patients, without ruling out their need in COVID-19-infected children.

The most important limitation of this study with a potential negative impact on the statistical analysis consists in the relatively small number of cases included in the two groups. Moreover, it might have been valuable to assess children from other geographic areas in order to identify potential risk factors associated with geographic or ethnic disparities. Nevertheless, our study is among the first to assess the discrepancies between COVID-19 and PIMS in children, and we consider that these findings might represent a major clinical asset for identifying the thin boundary between these two novel entities which burden the pediatrician’s daily practice.

## 5. Conclusions

COVID-19 and PIMS are two similar conditions, but yet very different in terms of outcome and prognosis. Albeit COVID-19 in children is most often a mild disease with favorable clinical course, PIMS can potentially result in short-term life-threatening complications and long-term invalidating sequelae. Therefore, it is crucial to distinguish these two clinical entities and establish a correct diagnosis, especially in PIMS patients that present a positive antigen or RT-PCR test. This study attempted to establish certain guidance criteria in order to ease the pediatrician’s work. Thus, we noticed that fever might be a clinical criterium in favor of PIMS. Moreover, hypoalbuminemia and increased ESR along with elevated D-dimers, NT-proBNP, and CK-MB might be considered reliable laboratory indicators of PIMS. Pathological findings at echocardiography also tilt the balance toward PIMS. Moreover, complications such as cardiac or liver impairment and dehydration are also more common in patients with PIMS, suggesting once more that PIMS is more severe than COVID-19, and its early diagnosis is essential for patient outcomes and follow-up. These findings represent the basis for further studies on larger samples in order to increase the accuracy of diagnosing these entities, since the COVID-19 pandemic is far from its end.

## Figures and Tables

**Table 1:** Correlation between COVID-19 or PIMS and gender or originating area.

Parameters	Group 1—COVID-19 (n = 46; %)	Group 2—PIMS (n = 20; %)	*p* Value
Female	22.00 (47.83%)	6.000 (30.00%)	0.28
Male	24.00 (52.17%)	14.00 (70.00%)	
Rural area	30.00 (65.22%)	11.00 (55.00%)	0.58
Urban area	16.00 (34.78%)	9.000 (45.00%)	

**Table 2:** The assessment of symptoms in the two groups.

Symptoms	Group 1—COVID-19 (n = 46; %)	Group 2—PIMS (n = 20; %)	*p* Value
Nasal obstruction	41.00 (89.13%)	15.00 (75.00%)	0.16
Dysphonia	8.000 (17.39%)	1.000 (5.000%)	0.26
Cough	22.00 (47.83%)	9.000 (45.00%)	0.99
Diarrhea	6.000 (13.04%)	3.000 (15.00%)	0.99
Vomiting	6.000 (13.04%)	6.000 (30.00%)	0.16
Abdominal pain	16.00 (34.78%)	10.00 (50.00%)	0.28
Myalgia	6.000 (13.04%)	6.000 (30.00%)	0.16
Fever	6.000 (13.04%)	8.000 (40.00%)	**<0.05**

**Table 3:** The assessment of hematological and biochemical parameters.

Parameters	Group 1—COVID-19 (n = 46; %) Means ± SD (Median Values)	Group 2—PIMS (n = 20; %) Means ± SD (Median Values)	*p* Value
ALT (UI)	63.26 ± 243.5 (17.050)	34.11 ± 36.27 (19.25)	* 0.23
AST (UI)	52.78 ± 75.79 (33.50)	46.36 ± 45.17 (26.45)	* 0.63
Total bilirubin (mg/dL)	0.6848 ± 0.6556 (0.5000)	0.3115 ± 0.1514 (0.2700)	* <0.05
Creatinine (mg/dL)	0.5848 ± 0.6345 (0.5000)	0.4030 ± 0.1589 (0.3500)	* <0.05
Urea (mg/dL)	27.79 ± 25.35 (21.40)	21.72 ± 6.945 (21.45)	* 0.69
Na (mmol/L)	139.0 ± 3.537 (139.0)	137.7 ± 3.856 (137.0)	* 0.16
K (mmol/L)	4.352 ± 0.5943 (4.300)	4.142 ± 0.6463 (4.120)	0.20
Total protein (g/dL)	6.472 ± 1.0800 (6.660)	6.344 ± 1.264 (6.330)	0.67
Albumin (g/dL)	4.509 ± 0.6616 (4.590)	3.455 ± 0.7075 (3.450)	* <0.05
LDH (U/L)	330.5 ± 122.0 (314.5)	281.9 ± 84.94 (256.5)	* 0.17
Leukocytes (×10^3^/µL)	11.25 ± 10.13 (8.800)	10.042 ± 5.271 (9.175)	* 0.90
Hgb (g/dL)	11.43 ± 2.224 (11.55)	11.34 ± 2.052 (10.90)	0.88
Htc (%)	34.45 ± 6.062 (35.05)	34.52 ± 5.705 (33.90)	0.96
VEM (pg)	79.59 ± 8.591 (80.75)	80.64 ± 7.814 (80.30)	* 0.67
Platelets (×10^3^/µL)	273.5 ± 121.4 (278.5)	335.4 ± 169.8 (309.5)	0.10
Neutrophils (×10^3^/µL)	5.428 ± 3.824 (4.570)	5.602 ± 4.248 (4.940)	* 0.98
Lymphocytes (×10^3^/µL)	4.843 ± 9.586 (2.540)	2.935 ± 1.567 (2.960)	* 0.80
Monocytes (number/µL)	1034 ± 720.0 (900.0)	1167 ± 817.5 (845.0)	* 0.59
Eosinophils (µL)	127.1 ± 269.4 (50.00)	342.3 ± 1133 (22.50)	* 0.92
ESR (mm/h)	17.39 ± 16.33 (10.00)	38.25 ± 35.54 (24.00)	* <0.05
CRP (mg/L)	32.87 ± 50.76 (6.800)	68.43 ± 72.99 (47.28)	* 0.08
Ferritin (ng/mL)	264.9 ± 650.6 (91.15)	200.6 ± 163.6 (177.8)	* 0.21

Legend: ALT—alanine aminotransferase, AST—aspartate aminotransferase, CRP—C-reactive protein, ESR—erythrocyte sedimentation rate, Hgb—hemoglobin, Htc – hematocrit, K—potassium, n—number, LDH—lactic dehydrogenase, Na- sodium, SD—standard deviation, VEM—medium erythrocyte volume, * Mann–Whitney test was used.

**Table 4:** The comparison between the inflammatory parameters.

Parameters	Group 1—COVID-19 (n = 46; %)			Group 2—PIMS (n = 20; %)			*p* Value
	Number (n) and %	Odds Ratio	95% Confidence Interval	Number (n) and %	Odds Ratio	95% Confidence Interval	
**IL-6 (pg/mL)**	8.000 (17.39%)	1.895	0.3640–9.849	2.000 (10.00%)	0.5278	0.1015–2.743	0.71
**D-dimers (ng/mL)**	12.00 (26.09%)	0.1176	0.0352–0.3936	15.00 (75.00%)	8.500	2.541–28.43	**<0.05**
**NT-proBNP (pg/mL)**	9.000 (19.57%)	0.2432	0.0778–0.7606	10.00 (50.00%)	4.111	1.315–12.85	**<0.05**
**hs-cTnI (pg/mL)**	2.000 (4.350%)	0.4091	0.0534–3.133	2.000 (10.00%)	2.444	0.3192–18.72	0.58
**CK-MB (U/L)**	6.000 (13.04%)	0.1227	0.0359–0.4199	11.00 (55.00%)	8.148	2.381–27.88	**<0.05**
**CK (U/L)**	9.000 (19.57%)	1.378	0.3306–5.746	3.000 (15.00%)	0.7255	0.1740–3.024	0.74

**Legend**: CK—creatine kinase, CK-MB—myocardial fragment of creatine kinase, hs-cTnI—transitioning high sensitivity cardiac troponin I, IL—interleukin, NT-proBNP—N-terminal pro Natriuretic Peptide type B.

**Table 5:** The logistic regression of predictors in COVID-19 versus PIMS groups.

Predictors	COVID = 19 (n = 46; %)			PIMS (n = 20; %)			*p* Value
	Exp(B)	95% C.I.for EXP(B)		Exp(B)	95% C.I.for EXP(B)		
		Lower	Upper		Lower	Upper	
**IL-6 (pg/mL)**	5.316	0.5160	54.71	0.1880	0.0180	1.936	0.16
**D-dimers (ng/mL)**	0.0560	0.0090	0.3460	17.96	2.890	111.7	**<0.05**
**NT-proBNP (pg/mL)**	2.014	0.3150	12.88	0.4960	0.0780	3.174	0.46
**hs-cTnI (pg/mL)**	0.2180	0.0110	4.384	4.583	0.2280	92.09	0.32
**CK-MB (U/L)**	0.1020	0.0200	0.5090	9.802	1.965	48.91	**<0.05**
**CK (U/L)**	7.635	0.9700	60.08	0.1310	0.0170	1.031	0.05

**Table 6:** Comparison between crude and adjusted OR.

Predictors	COVID-19						PIMS						*p* Value for Ad-justed OR	*p* Value for Crude OR
	Adjusted OR	95% C.I.for Adjusted OR		Crude OR	95% C.I.for Crude OR		Adjusted OR	95% C.I.for Adjusted OR		Crude OR	95% C.I.for Crude OR			
		Lower	Upper		Lower	Upper		Lower	Upper		Lower	Upper		
**IL-6 (pg/mL)**	5.316	0.5160	54.71	1.895	0.3640	9.849	0.1880	0.0180	1.936	0.5278	0.1015	2.743	0.16	0.71
**D-dimers (ng/mL)**	0.0560	0.0090	0.3460	0.1176	0.0352	0.3936	17.96	2.890	111.7	8.500	2.541	28.43	**<0.05**	**<0.05**
**NT-proBNP (pg/mL)**	2.014	0.3150	12.88	0.2432	0.0778	0.7606	0.4960	0.0780	3.174	4.111	1.315	12.85	0.46	**<0.05**
**hs-cTnI (pg/mL)**	0.2180	0.0110	4.384	0.4091	0.0534	3.133	4.583	0.2280	92.09	2.444	0.3192	18.72	0.32	0.58
**CK-MB (U/L)**	0.1020	0.0200	0.5090	0.1227	0.0359	0.4199	9.802	1.965	48.91	8.148	2.381	27.88	**<0.05**	**<0.05**
**CK (U/L)**	7.635	0.9700	60.08	1.378	0.3306	5.746	0.1310	0.0170	1.031	0.7255	0.1740	3.024	0.05	0.74

**Table 7:** Associated complications of the two groups.

Diseases	Group 1—COVID-19 (n = 46; %)	Group 2—PIMS (n = 20; %)	*p* Value
Cardiac impairment	7.000 (15.22%)	13.00 (65.00%)	**<0.05**
Liver impairment	9.000 (19.57%)	11.00 (55.00%)	**<0.05**
Renal impairment	7.000 (15.22%)	0.0000 (0.0000%)	**-**
Skin impairment	2.000 (4.350%)	3.000 (15.00%)	0.16
Respiratory complications	22.00 (47.83%)	7.000 (35.00%)	0.42
Gastrointestinal disease	9.000 (19.57%)	2.000 (10.00%)	0.48
Dehydration syndrome	5.000 (10.87%)	10.00 (50.00%)	**<0.05**
Encephalitis	2.000 (4.350%)	2.000 (10.00%)	0.58
Polyradiculoneuritis	0.0000 (0.0000%)	1.000 (5.000%)	-
Stroke	0.0000 (0.0000%)	1.000 (5.000%)	-

## Data Availability

The data presented in this study are available on request from the corresponding author.
